# The combined association of depression and socioeconomic status with length of post-operative hospital stay following coronary artery bypass graft surgery: Data from a prospective cohort study^☆^

**DOI:** 10.1016/j.jpsychores.2013.10.019

**Published:** 2014-01

**Authors:** Lydia Poole, Elizabeth Leigh, Tara Kidd, Amy Ronaldson, Marjan Jahangiri, Andrew Steptoe

**Affiliations:** a Department of Epidemiology and Public Health, University College London, 1-19 Torrington Place, London, UK; b Department of Cardiac Surgery, St George's Hospital, University of London, Blackshaw Road, London, UK

**Keywords:** ACS, acute coronary syndrome, ARCS study, Adjustment and Recovery after Cardiac Surgery study, BDI, Beck Depression Inventory, BMI, body mass index, CABG, coronary artery bypass graft, CI, confidence intervals, EuroSCORE, European System for Cardiac Operative Risk Evaluation, HADS, Hospital Anxiety and Depression Scale, Depression, Coronary artery bypass graft surgery, Recovery, Length of stay, Income, Socioeconomic position

## Abstract

**Objective:**

To understand the association between pre-operative depression symptoms, including cognitive and somatic symptom subtypes, and length of post-operative stay in patients undergoing coronary artery bypass graft (CABG) surgery, and the role of socioeconomic status (SES).

**Methods:**

We measured depression symptoms using the Beck Depression Inventory (BDI) and household income in the month prior to surgery in 310 participants undergoing elective, first-time, CABG. Participants were followed-up post-operatively to assess the length of their hospital stay.

**Results:**

We showed that greater pre-operative depression symptoms on the BDI were associated with a longer hospital stay (hazard ratio = 0.978, 95% CI 0.957–0.999, *p* = .043) even after controlling for covariates, with the effect being observed for cognitive symptoms of depression but not somatic symptoms. Lower SES augmented the negative effect of depression on length of stay.

**Conclusions:**

Depression symptoms interact with socioeconomic position to affect recovery following cardiac surgery and further work is needed in order to understand the pathways of this association.

## Introduction

Depression is prevalent among coronary artery bypass graft (CABG) patients with pre-surgery estimates ranging between 14.3% and 43.1% [1–5]. Patients with pre-operative depression have been found to experience a host of poorer surgical recovery outcomes including higher incidence of medical complications during the six months following surgery, and greater reporting of poor quality of life and worse recovery [2]. One important marker of surgical recovery is length of post-operative hospital stay. In recent decades length of post-operative hospital stay has reduced significantly after CABG. Many institutions in the UK apply an ‘early discharge’ protocol whereby the aim is to discharge elective patients within five days. While post-operative stay is a proxy measure of acute physical recovery, it is also an important indicator of recovery in the longer term, as it has been found to be associated with hospital readmission [6] and recurrent cardiac events [7]. Many clinical risk factors are established predictors of prolonged length of stay [8], though interest is now being paid to psychosocial risk factors including the role of pre-operative depression [9]. This latter study found that greater pre-operative depression symptoms, in a sample of 117 elective CABG patients, were associated with longer hospital stays after controlling for demographic and clinical risk factors.

The type of depression symptoms experienced by patients has also been studied in CABG patients, but research has tended to focus on cardiac prognosis rather than acute recovery. For example, Connerney and colleagues [7] found cognitive/affective but not somatic symptoms to be predictive of cardiac mortality in models adjusted for confounders. These results have been supported by another recent study of CABG patients [10]. These findings are in contrast to data concerning acute coronary syndrome (ACS) patients [11–16] and therefore warrant further investigation. This issue is of particular salience to acute recovery endpoints since somatic symptoms of depression may overlap with the physical symptoms of illness; therefore by investigating the unique contribution of both depression symptom subtypes we may be able to tease out the effect of depression on cardiac health and minimise any residual confounding.

Socioeconomic status (SES) has been implicated in the link between depression and coronary heart disease. In a sample of 298 men and women low household income was associated with elevated depression symptoms at three weeks, six months and one year following ACS and this effect was independent of other demographic and clinical risk factors as well as history of depression [17]. In CABG samples, some investigators have used another marker of socioeconomic position, education, finding that low education is associated with greater risk of pre-operative depression [4,18], but others have not supported this effect [2]. However, educational attainment may not be the optimal marker of SES in this patient group, since most have completed formal education several years before the onset of cardiac disease. Therefore, measures of current socioeconomic resources may be more appropriate.

Little is known about the interaction between depression and SES for CABG recovery. However, population research has shown that low occupational status acts synergistically with psychological distress to impact cardiovascular mortality negatively [19]. The reason for this is not clear, but a biological mechanism is plausible in which depression and low SES act as stressors, leading to wear and tear on the body in line with the allostatic load model of stress [20,21]. This notion is particularly pertinent when one considers recent evidence of the distinct biological correlates of cognitive versus somatic depression, in which cognitive depression is thought to align itself with changes to the neuroendocrine system, whereas somatic depression is thought to be more closely related to inflammation [22]. Such a hypothesis would be consistent with the notion that somatic depression and the physical symptoms of illness are inextricably linked.

The aim of this study was to assess the relationship between pre-operative depression symptoms and length of post-operative stay, and to examine the extent to which any association could be influenced by differences in SES. Specifically, we hypothesised that cognitive and somatic symptoms of depression would differentially be associated with length of hospital stay, and that the effect of depression symptoms on length of stay would be moderated by household income.

## Methods

### Participants

**Fig. 1:**
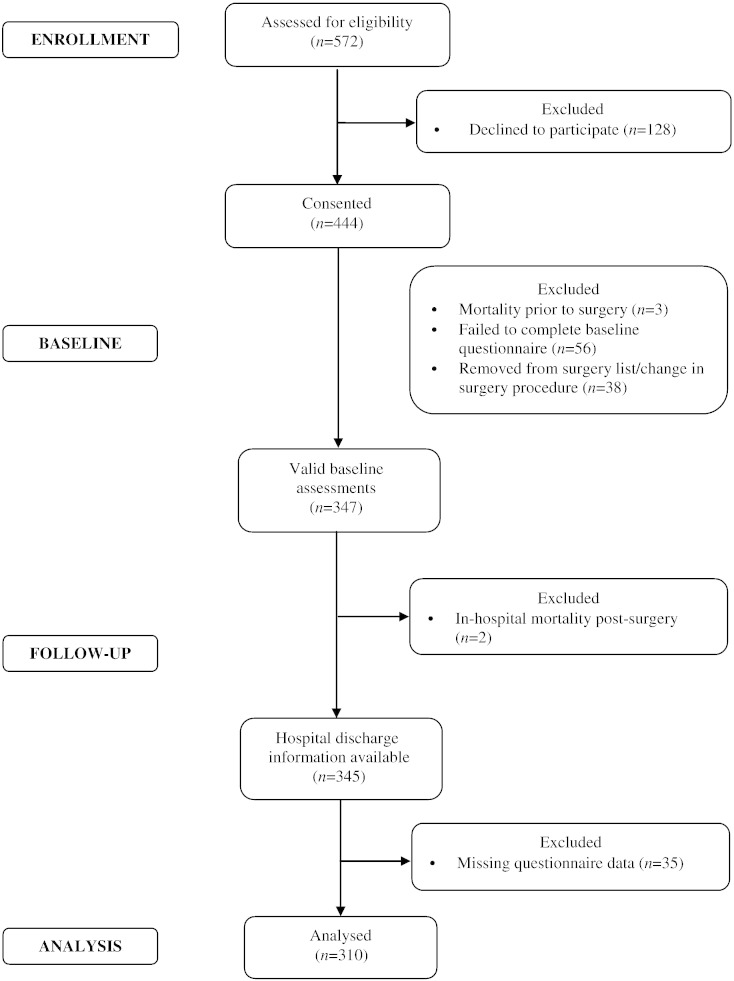
Flow diagram of participant recruitment and attrition.

The study uses data collected in the Adjustment and Recovery after Cardiac Surgery (ARCS) study which was designed to investigate the causes and consequences of poor emotional adjustment following cardiac surgery and was powered to assess the importance of a broad range of psychological, behavioural and social factors. The recruitment and retention of participants into the ARCS study is displayed in Fig. 1. Briefly, out of the 347 participants who completed valid baseline questionnaires, those participants included in these analyses were the 310 CABG surgery patients (mean age: 67.76 ± 9.17 years, 14.8% females) with complete data for all variables at baseline and follow-up, including covariates. Compared to the participants who were included in these analyses, the excluded participants were more likely to be female (*x*^2^(1) = 5.34, *p* = .021), but otherwise did not differ on any other clinical or demographic variable. Participants were recruited consecutively from a pre-surgery assessment clinic at St. George's Hospital, London, between January 2010 and July 2012. The baseline assessment took place on average 29 days before patients' surgery when they came to the hospital for their pre-assessment clinic appointment. Inclusion criteria permitted only patients who were undergoing elective CABG surgery or CABG plus valve replacement to participate. CABG surgery was defined to include both on-pump and off-pump surgical procedures. In addition, participants had to be able to complete the questionnaires in English, and be 18 years or older. All procedures were carried out with the written consent of the participants. Ethical approval was obtained from the South West London research ethics committee.

### Measures

#### Predictors: depression and SES measures

The Beck Depression Inventory (BDI) [23] was used to measure depression symptoms at baseline. It is a 21-item questionnaire which asks the respondent to reflect on how they have been feeling over the past two weeks. Ratings were summed, with higher scores indicating greater emotional disturbance, with a range of 0 to 63 (Cronbach's α = 0.85). A severity categorical variable was generated according to accepted cut-offs: a score of 0 to 10 indicating no depression, 11 to 20 mild depression, and 21 and above moderate to severe depression. We also generated two symptom subtype scores as described by Beck and Steer [24]. The cognitive depression symptom score was the sum of items 1 to 13 and the somatic depression symptom score was the sum of items 14 to 21. SES was assessed using yearly household income divided into five categories ranging from <£10,000 per year to >£40,000 per year.

#### Outcome: length of stay measure

Length of post-operative hospital stay was collected from clinical records. The discharge policy at St. George's Hospital is to discharge all patients within 7 days of CABG providing there are no complications; there were no changes to this discharge policy during the period of data collection. Length of stay is a marker of clinical recovery, with those participants experiencing the poorest recovery and the greatest in-hospital complications, expected to have the longest hospital stays after CABG.

#### Covariates: clinical and sociodemographic measures

Clinical risk was assessed using the European System for Cardiac Operative Risk Evaluation (EuroSCORE) [25]. EuroSCORE is a composite measure of procedural mortality risk based on 17 factors comprising patient-related factors (e.g. age, sex), cardiac-related factors (e.g. unstable angina, recent MI) and surgery-related factors (e.g. surgery on thoracic aorta). Items were scored in accordance with the ‘logistic EuroSCORE’ method to generate a percentage mortality risk estimate; further details of the scoring method can be found on the EuroSCORE website (www.euroscore.org/logisticEuroSCORE.htm) [26]. In addition, the number of grafts a participant received and whether they underwent cardiopulmonary bypass (yes/no) were also recorded. History of diabetes was also taken from medical notes, with participants being categorised according to their treatment status: none, diet, oral hypoglycaemic drugs or insulin. Cardiovascular history, clinical factors during admission and management were also obtained from clinical notes.

Participants were asked to self-report any longstanding illnesses prior to surgery; responses were counted to compute a chronic illness burden variable to capture the number of illnesses a participant had in addition to their coronary artery disease. Prescribed medication use was recorded, including use of antidepressants. Smoking was measured as a binary variable (current smoker/non-smoker). Body mass index (BMI) was assessed at the pre-operative clinic appointment and calculated using the standard formula (kg/m^2^).

The Hospital Anxiety and Depression Scale (HADS) is a self-report measure of anxiety and depression for use in outpatient clinical settings [27]. Only the 7-item anxiety scale was administered at baseline, capturing the extent to which each symptom had been experienced over the past two weeks. Items were summed to generate an overall score, with higher scores indicating greater anxiety (Cronbach's α = 0.89).

### Statistical analysis

Associations between variables were assessed using Pearson's correlations for continuous data and one-way ANOVAs for categorical variables. To test the association between baseline depression and length of hospital day, we analysed baseline depression as a continuous variable, and modelled associations between pre-operative depression and post-operative length of stay using Cox proportional hazards regressions. The dependent variable in these analyses was whether the patient had been discharged from hospital (coded 1) or remained in hospital (coded 0) on every day following surgery; consequently, a hazard ratio < 1.0 indicates a longer length of stay. Since length of stay data were available for all participants included in these analyses, the results are modelled on the entire sample. We included covariates that might potentially relate to the outcomes including: BMI, smoking status, household income, diabetes status, chronic illness burden, cardiopulmonary bypass, number of grafts, EuroSCORE, depression medication, and anxiety. Since EuroSCORE includes age and sex in the score, these variables were not included in the fully adjusted model to avoid double adjustment. Models adjusting for use of statin medications were also performed, but are not reported here since the results did not change; statin records were missing for 32 participants. Results are presented as adjusted hazard ratios with 95% confidence intervals (CI). Secondary Cox regression analyses were performed using the BDI severity categorical variable and the cognitive and somatic BDI scores split into tertiles to predict length of stay. To avoid over-fitting of these categorical models we adjusted for age and sex only here. These results confirmed that the assumption of proportional hazards was upheld in all the models. We illustrated the significant associations by comparing the length of stay outcome in patients in the depression severity groups.

We tested the combined association between length of hospital stay and pre-operative depression and household income by dividing the sample into ‘no’ and ‘high’ depression subgroups using the standard BDI cut-off of 10 to indicate mild to moderate depression, and a median split on income at £20,000/year. We used these binary variables to create four categories of participants: no depression symptoms/high household income, no depression symptoms/low household income, high depression symptoms/low household income, and high depression symptoms/high household income. The hazard of rate of hospital stay were calculated in these four groups, using the no depression symptoms/high household income as reference, and adjusting for BMI, smoking status, diabetes status, chronic illness burden, cardiopulmonary bypass, number of grafts, EuroSCORE, depression medication and anxiety, and are presented as adjusted hazard ratios with 95% CI. We chose not to model this interaction using continuous variables since this would combine a continuous variable (BDI) with a categorical variable (household income), which would likely lead to an imbalance between the relative impact of the two on the interaction term. All analyses were conducted using SPSS version 21.

## Results

Table 1 summarises the characteristics of the participants at baseline, prior to CABG surgery. The sample had an age range between 22 and 90 years, was predominantly male (85.2%) and overweight (BMI > 25 = 81.6%). The majority of participants were hypertensive, and approximately a quarter of patients were diabetic. The majority of participants had on-pump cardiopulmonary bypass surgery in isolation. The average length of post-operative hospital stay was 7 days, with a range of 4 to 66 days. On average participants were within the normal range for depression symptoms on the BDI, however 94 (30.3%) participants scored > 10 at baseline. Greater depression symptoms were associated with younger age (*r* = − 0.184, *p* = 0.001), female sex (*t*(308) = − 2.555, *p* = 0.011), lower household income (*r* = − 0.130, *p* = 0.022), smoking (*t*(308) = − 3.719, *p* < 0.001), higher BMI (*r* = 0.160, *p* = 0.005), greater anxiety (*r* = 0.622, *p* < 0.001) and hypertension (*t*(308) = − 2.121, *p* = 0.035). No associations were found between BDI scores and use of statin or antidepressant medication. Longer hospital stays were associated with older age (*r* = 0.227, *p* < 0.001), female sex (*t*(308) = − 2.957, *p* = 0.003), lower household income (*r* = − 0.143, *p* = 0.012), greater chronic illness burden (*r* = 0.115, *p* = 0.044) and greater EuroSCOREs (*r* = 0.243, *p* < 0.001).

#### The association between depression symptoms and length of stay

**Fig. 2:**
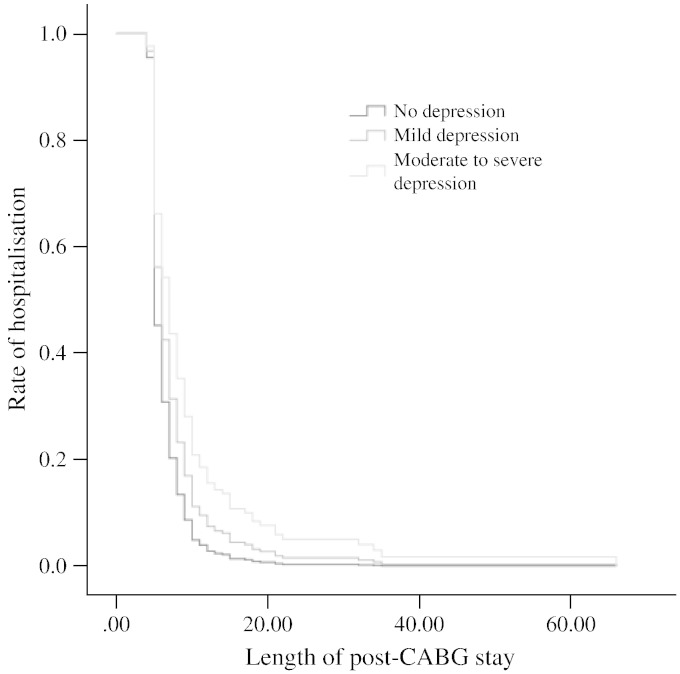
Length of post-operative hospital stay by depression severity status on the BDI; age and sex adjusted model.

Cox regression models predicting the rate of hospitalisation for the total, cognitive and somatic depression symptom scores on the BDI are displayed in Table 2. Total BDI scores were a significant predictor of longer hospital stays in the fully adjusted model (*p* = 0.043). EuroSCORE was the only other significant predictor in this model (hazard ratio = 0.912, CI = 0.872–0.954, *p* < 0.001). Cognitive depression scores were also a significant predictor of longer post-operative stays (*p* = 0.037) with the only other significant predictors in the fully adjusted models being EuroSCORE (hazard ratio = 0.908, CI = 0.868–0.9950, *p* < 0.001) and household income (hazard ratio = 1.095, CI = 1.007–1.190, *p* = 0.033). Somatic symptoms of depression were associated with length of stay after controlling for age and sex (*p* = 0.041), but not in the fully adjusted model (*p* = 0.192). Additional adjustment for use of statins did not alter the results (results not presented here). For total BDI, the three depression severity groups of none, mild and moderate to severe, corresponded to 6.95, 7.44, and 8.33 post-operative days in hospital respectively. The continuous results were confirmed using the categorical BDI variables (see Table 3) in which the depression severity categorical variable and the cognitive depression symptom tertile variable were both significantly associated with length of stay. The association between the highest somatic depression tertile and length of stay approached significance in the age and sex adjusted model (*p* = 0.057), and was not significant after all the covariates had been taken into account (results not shown). This association is represented in Fig. 2.

#### The interaction between depression and SES to predict length of stay

We assessed the interaction between depression symptoms and household income in Cox regression models adjusting progressively for covariates (Table 4). Compared with the reference group of no depression symptoms/high household income, patients in the high depression symptoms/low household income group had significantly longer hospital stays (*p* = 0.006). This was not the case for the high depression symptoms/high household income group, suggesting high income was associated with a reduction in the negative effect of depression symptoms on length of stay after surgery. This pattern of results was replicated using the cognitive depression symptom data; such that those participants in the high cognitive depression symptoms/low household income group (hazard ratio = 0.620, CI = 0.432–0.891, *p* = 0.010) had significantly longer hospital stays compared to the reference group of no cognitive depression symptoms/high household income group in a fully adjusted model.

## Discussion

In the present study we aimed to study the prospective association between pre-operative depression symptoms and post-operative length of hospital stay in a sample of participants undergoing elective CABG surgery. We showed that greater depression symptoms were associated with longer hospital stays. In fact, a dose–response relationship between depression symptoms and length of stay was found, with those participants with mild or moderate to severe depression symptoms being at risk of a longer stay compared to those with no depression symptoms. In depression symptom subtype analyses, we showed that the cognitive symptoms of depression, but not the somatic symptoms, were associated with a greater length of stay. Finally, we also showed an interaction between depression symptoms and household income, such that having lower income augmented the negative effect of depression, while a higher income was associated with a reduction in the negative effect of depression symptoms on length of stay.

Our findings are in line with others who have noted that depression symptoms are prevalent before CABG surgery. One third of participants had elevated scores on the BDI pre-operatively. Prevalence of depression before CABG surgery appears to vary widely, with other studies reporting rates between 14.3% and 43.1% [1–5]. Inconsistencies in these estimates are likely due to differences in the method of depression assessment, different uses of cut-offs on questionnaire measures, differences in the timing of assessment and demographic differences between samples. Moreover, since the late 1990s the UK has seen a fifteen-fold increase in the prescription of lipid-lowering medications [28]. These differences in pre-surgical medication use may partly account for differences in depression prevalence since there is some evidence for the depression-protective effects of statins [29,30]. Furthermore, the use of antidepressant medication has not been consistently taken into account, and this may have led some studies to underestimate the prevalence of depressive symptoms. In our sample, we found no relationship between depression symptoms on the BDI and use of statin or antidepressant medication.

The average length of stay in our sample was 7.15 days, which is consistent with the hospital protocol where our participants were recruited. In a study which aggregated length of stay data between 2007 and 2009 across 28 UK hospitals, the mean stay for the 19,522 CABG patients sampled was found to be 12.48 days (standard deviation = 10.94) [31]. It is likely that the fact we recruited only elective, first-time CABG patients largely accounts for the shorter stays reported here. We showed both mild and moderate to severe pre-operative depression symptoms to be associated with longer post-operative hospital stays; models using continuous BDI scores supported the linear relationship. Greater pre-operative depression symptoms have previously been associated with longer hospital stays after controlling for demographic and clinical risk factors [9]; however, little research has studied the effects of depression symptom subtypes in CABG patients.

The depression symptom profile of cardiac patients has been found in some studies to be differentially predictive of prognosis. Somatic symptoms have found to be particularly damaging following ACS [11–16]. Among studies investigating CABG patients, depression symptom subtypes in relation to recovery have been little studied and the results have generally not tallied with the ACS studies. For example, Connerney and colleagues [32] found cognitive/affective but not somatic symptoms to be predictive of cardiac mortality. These results have been supported by another study of morbidity and mortality in CABG patients, which also found cognitive depressive symptoms to be particularly damaging [10]. Our results are in line with these findings, suggesting that the cognitive symptoms of depression are particularly important for predicting increased length of hospital stay in CABG patients. However, the hazard ratio for cognitive depression symptoms predicting length of stay was 0.965, whereas for somatic depression symptoms it was 0.973, reflecting only a small difference in the overall size of the effect. Therefore, care must be taken when interpreting these results. It is also worth noting that somatic depression symptoms were a significant predictor of length of stay in age and sex adjusted models, but not in fully adjusted models. This might suggest that somatic symptoms of depression are confounded with comorbidities and clinical features, rather than having independent significance. It is not clear why ACS and CABG surgery should have these differences in the sequelae of the depression symptom subtypes, but issues surrounding differences in the clinical characteristics of participants may partly contribute to the discrepancy. For example, the cardiac function over the recovery period may be more closely related to somatic than to cognitive symptoms of depression. The difference may also be related to the phenomenology of the experience. CABG is planned, somewhat predictable, and optional (the patient can choose whether to have surgery), while on the other hand an ACS is unplanned, unexpected, unpredictable and uncontrollable. From a clinical perspective, these results suggest that strategies aimed to improve and treat depression as a homogenous disorder, and according to current diagnostic criteria, may not necessarily confer therapeutic benefits; more work is needed to address this issue.

We explored the role of socioeconomic status in the relationship between depression and length of stay, finding that while household income was not an independent predictor in regression models, it did interact with depression symptoms to affect the outcome. Specifically, we found that those participants with both high depression symptoms and low income were at risk of longer hospital stays than those with no depression symptoms and high income; in fact they stayed almost two days more (8.32 days versus 6.21 days respectively). This represents a clinically significant result when one considers the economic implications for the hospital as well as the pressure for bed spaces for incoming patients. Weintraub and colleagues [33] estimated the cost of hospital stays for CABG surgery to range between £976.62 for every day spent in intensive care and £169.49 for every day spent in the general ward. It is important to note that the surgery was carried out in a public hospital, where issues such as insurance cover and cost do not influence length of stay. Furthermore, higher income seemed to have a buffering effect on depression, such that patients reporting high levels of depression symptoms and who also had a high income were not at increased risk of a longer stay. These findings are in line with previous research which has shown the importance of SES in cardiac patients. In particular, they are consistent with work by Lazzarino and colleagues [19] who reported socioeconomic status interacted with psychological distress, such that low SES compounded the negative effect of distress on cardiac mortality. Our results are the first data that we are aware of to show that high income was associated with a reduction in the detrimental effects of depression symptoms in CABG patients.

The mechanisms linking depression and SES to longer hospitalisation following CABG surgery are not clear, but are likely to include multiple pathways such as impaired wound healing, greater susceptibility to infection, slow resolution of inflammatory responses, and impaired behavioural adaptation. Such factors have been reviewed in relation to depression [34] and also SES [35]. Further work is needed to delineate these biological and behavioural pathways in CABG patients. The direction of the interaction between depression and SES also requires further study. One possibility is that people from higher SES backgrounds are more likely to have better social and financial resources to enable them to engage in adaptive coping strategies, protecting them against depression and its negative sequelae [36]. A simple mechanism should also not be overlooked, in which low SES depressed CABG patients are less likely to be discharged within 7 days due to concerns surrounding the suitability of the home environment.

There are several strengths to our study. The prospective association between pre-operative depression and post-operative hospital stays allows the direction of the effect to be explored. In addition, our analyses controlled for a large range of potential confounders, including clinical, demographic and anxiety factors, still demonstrating an effect of depression on length of stay. Another strength is that the ARCS study examined patients undergoing CABG at a single hospital and therefore removes the influence of inter-hospital variation in discharge policy. However, a number of limitations also need to be borne in mind. Firstly is the reliance on questionnaire measures of emotional distress, including depression symptoms and anxiety. Questionnaire measures capture depression symptoms and not clinical depression per se. Secondly, while we studied the effect of one marker of SES in this study, other markers were not investigated such as education and ethnicity. While household income is a contemporaneous measure of SES not all participants disclosed this information (n = 30) leading to missing data in our sample. As with many cohort studies, we coded income as a categorical variable in the ARCS study which renders it difficult to generalise our findings across samples and across time. In addition, care needs to be taken with the interpretation of length of stay, since it is only a proxy marker of physical recovery; other factors such as social housing constraints may influence discharge times of some patients. Our results have clear clinical implications surrounding the screening and treatment of depression symptoms prior to CABG surgery. However, more work is needed in order to investigate the best ways to approach the treatment of depression in this patient group and to ensure alleviation of depression symptoms translates to improvements in surgical recovery.

In conclusion, we have found that pre-operative depression symptoms were associated with longer post-operative hospital stays in patients undergoing CABG surgery, but that higher household income was associated with a reduction in this negative effect. Further work is needed in order to understand the processes through which depression and socioeconomic status interact to affect cardiac recovery.

## Funding

This research was funded by the British Heart Foundation.

## Conflict of interest

The authors have no conflicts of interest to declare.

## Figures and Tables

**Table 1:** Demographic, clinical and depression characteristics of the sample at baseline (N = 310)

Characteristic	Mean ± SD or N (%)
Age (years)	67.76 ± 9.17
Female	46 (14.8)
BMI (kg/m^2^)	28.64 ± 4.23
Married/cohabiting	227 (73.2)
Ethnicity — White British/Other White	274 (88.4)
Yearly household income	
< 10,000 GBP	60 (19.4)
10,000–20,000 GBP	95 (30.6)
20,000–30,000 GBP	62 (20.0)
30,000–40,000 GBP	44 (14.2)
> 40,000 GBP	49 (15.8)
Smoker	25 (8.1)
Statin medication	241 (86.7)^a^
Antidepressant medication	15 (4.8)
Anxiety (HADS subscale)	6.01 ± 4.41

*Co-morbidities*	
Diabetes	77 (24.8)
Hypertension	247 (79.7)
Pulmonary disease	25 (8.1)
Neurological disorder	28 (9.0)
Extracardiac arteriopathy	25 (8.1)
Chronic illness burden	0.36 ± 0.64

*Clinical factors*	
Logistic EuroSCORE (%)	4.20 ± 2.95
CABG in isolation	240 (77.4)
Number of grafts	3.01 ± 1.07
On-pump	239 (77.1)
Length of post-operative hospital stay	7.15 ± 5.16

*Depression symptoms*	
Total BDI score	8.68 ± 6.61
Cognitive symptom score	3.48 ± 4.19
Somatic symptom score	5.22 ± 3.20
Depression severity	
None (0–10)	216 (69.7)
Mild (11–20)	79 (25.5)
Moderate to severe (21–63)	15 (4.8)

a N = 278.

**Table 2:** Depression symptoms (continuous BDI scores) and length of post-operative hospital stay

Model	HR	95% CI	*p*
*Total BDI score*			
Age and sex adjusted	0.975	0.958–0.993	.01
Fully adjusted^a^	0.978	0.957–0.999	.04

*Cognitive BDI score*			
Age and sex adjusted	0.959	0.931–0.988	.01
Fully adjusted^a^	0.965	0.933–0.998	.04

*Somatic BDI score*			
Age and sex adjusted	0.963	0.929–0.998	.04
Fully adjusted^a^	0.973	0.933–1.014	.19

a Fully adjusted model: household income, smoking status, BMI, diabetes, chronic disease burden, cardiopulmonary bypass, number of grafts, EuroSCORE, antidepressant medication and anxiety.

**Table 3:** Depression symptom categories and length of post-operative hospital stay

Model	Depression symptom level	HR	95% CI	*p*
*Total BDI score*				
Age and sex adjusted	None	–	1 (reference)	–
	Mild	0.726	0.552–0.955	.02
	Moderate to severe	0.519	0.300–0.899	0.02

*Cognitive BDI score*				
Age and sex adjusted	Low	–	1 (reference)	–
	Medium	0.762	0.577–1.008	.06
	High	0.701	0.525–0.935	.02

*Somatic BDI score*				
Age and sex	Low	–	1 (reference)	–
	Medium	0.906	0.693–1.185	.47
	High	0.753	0.563–1.008	.06

**Table 4:** Depression, yearly household income and length of post-operative hospital stay

Model	N	Mean length of stay (days)	HR	95% CI	*p*
No depression/high income	114	6.21	–	1 (Reference)	–
No depression/low income	102	7.78			
Age and sex adjusted			0.788	0.599–1.036	.09
Fully adjusted^a^			0.801	0.606–1.058	.12
High depression/high income	41	6.63			
Age and sex			0.735	0.505–1.071	.11
Fully adjusted^a^			0.810	0.540–1.215	.31
High depression/low income	53	8.32			
Age and sex			0.547	0.389–0.770	< .01
Fully adjusted^a^			0.599	0.415–0.864	.01

a Fully adjusted model: smoking status, BMI, diabetes, chronic disease burden, cardiopulmonary bypass, number of grafts, EuroSCORE, antidepressant medication and anxiety.
